# Menthol Toxicity: An Unusual Cause of Coma

**DOI:** 10.1155/2012/187039

**Published:** 2012-12-03

**Authors:** Motaz Baibars, Simona Eng, Khaldoon Shaheen, Abdul Hamid Alraiyes, M. Chadi Alraies

**Affiliations:** ^1^Hospital Medicine/Internal Medicine Department, Peninsula Regional Medical Center, Salisbury, MD 2180, USA; ^2^Department of Hospital Medicine, Cleveland Clinic, Cleveland, OH 44195, USA; ^3^Department of Pulmonary, Critical Care and Environmental Medicine, Tulane University Health Sciences Center, New Orleans, LA 70112, USA; ^4^Cleveland Clinic Lerner College of Medicine of Case Western Reserve University and Department of Hospital Medicine Institute, Cleveland Clinic, 9500 Euclid Avenue, Mail Code A13, Cleveland, OH 44195, USA

## Abstract

The US Food and Drug Administration (FDA) consider menthol an effective substance, which help in common cold symptoms and labeled to have low toxicity profile. Direct exposure to high menthol amount has been reported in animal; but no studies have been done to show the effect of menthol on long-term use in humans. Up to our knowledge we are reporting a rare case of chronic exposure to significant amount of menthol associated with cutaneous, gastrointestinal and neurological manifestations.

## 1. Introduction

The US Food and Drug Administration (FDA) considers menthol an effective substance, which help in common cold symptoms and labeled to have low toxicity profile. Direct exposure to high menthol amount has been reported in animal; but no studies have been done to show the effect of menthol on long-term use in humans. Up to our knowledge we are reporting a rare case of chronic exposure to significant amount of menthol associated with cutaneous, gastrointestinals and neurological manifestations.

## 2. The Case

**Figure 1:**
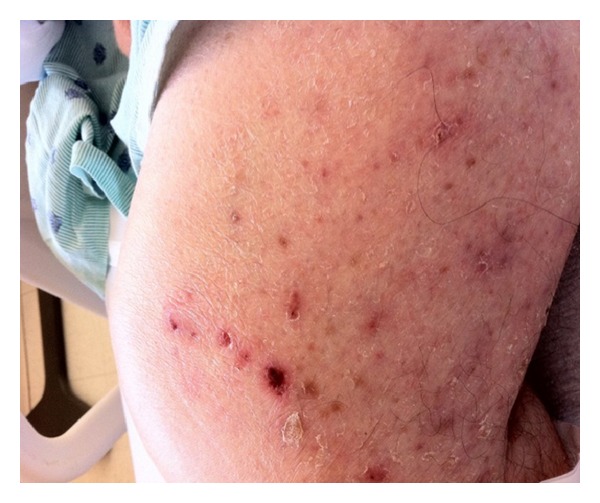


**Figure 2:**
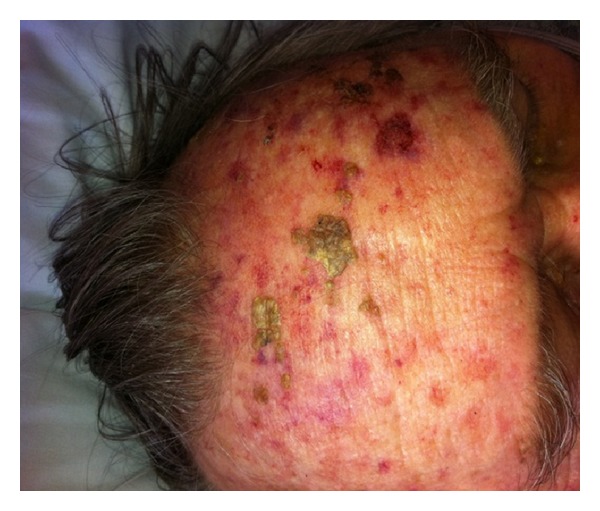


**Figure 3:**
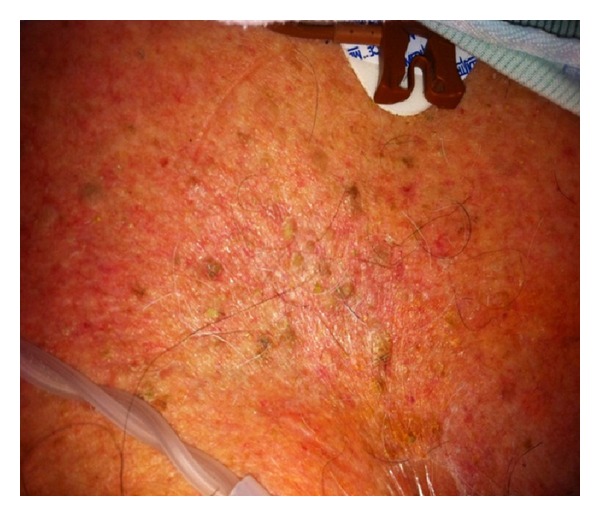


An 86-year-old living alone man presented to the emergency department after being found unresponsive by neighbors. On arrival, he was unresponsive but started to regain consciousness. He was confused with no recollection to what happened. He was complaining of weakness and generalized muscle aches. On further questioning he denied headaches, visual disturbance, motor, or sensory symptoms. He never had similar symptoms before. He takes no prescription medications since he does not have any reported medical problems and never saw a primary care physician ever before. He denied smoking, alcohol, or illicit drug use. Review of systems was significant for heartburn, recurrent oral aphthous ulcers, intermittent diarrhea, chronic dizziness, and unsteadiness while walking. On examination, vital signs were remarkable for blood pressure of 166/94 mmHg. He was disoriented to time and place, but able to follow simple commands. Skins examination showed multiple nontender, macular skin lesions in different sizes ranging 1–3 cm in diameter. These lesions were covered with dry yellow crusts over the forehead, scalp, trunk, and extremities (Figures 1, 2, and 3). Later the patient admitted for having these lesions for years. Neurological examination was with no focal deficits, but he had generalized decreased muscle strength. Gait was unsteady and required assistance. His chest and cardiovascular examination was unremarkable.

Blood work showed WBC of 23,000 cells/*μ*L, potassium 5.5 mmol/L, creatinine 2.64 mg/dL, BUN 42 mg/dL, anion gap 16 mmol/L, AST 2423 U/L, ALT 508 U/L, total bilirubin 2.1 mg/dL mainly indirect (1.8 mg/dL), and Creatinine Kinase (CK) of 42,000 mg/dL. Rhabdomyolysis was presumed due to prolonged immobilization and possible postictal state. CT scan of the head did not disclose any significant findings. Urine toxicology and serum alcohol levels were negative. He was admitted to the hospital and started on intravenous fluids. Liver enzymes were thought to be elevated due to massive CK elevation, which improved with fluids. Renal function did not improve, and patient continued to be oliguric with dark brown colored urine. Acute tubular necrosis (ATN) was confirmed and hemodialysis started. The patient was started on proton pump inhibitors for heartburn that gave him relieve. He had slow recovery and became more alert and oriented. He was able to stand up with significant ataxia. Further investigations including TSH, B12, EEG, brain MRI, CSF, RPR, and blood cultures were unremarkable.

In the absence of an obvious cause for coma and ataxia, further detailed history was obtained including dietary habits. The patient admitted for daily ingestion of two bags of menthol-rich cough droplet for twenty years. He has been taking these droplets mainly for halitosis. The amount of menthol the patient ingested was hard to determine. However, based on the brand he was ingesting, menthol concentration was 10 mg per droplet. He denied taking any other over the counter medications, supplements, or herbal medicine. 

During hospitalization, the patient continued to improve clinically with total regain of his mental function, but was unable to maintain his daily activities. He was discharged to rehabilitation for physical therapy. His kidney function recovered partially but remained on hemodialysis. He was counseled not to ingest menthol droplets anymore. After discharge, he had follow-up appointments with a primary care at 4, 8, and 12 weeks. His skin lesions, GI, and neurological symptoms disappeared and regained his total function 6 months after stopping menthol droplets.

## 3. Discussion

The FDA generally considers menthol; the primary constituent of peppermint oil, a safe substance, and toxicity is rarely reported. The Natural standards, a medical integrative international organization, mentioned a dose of up to 1 gram per kilogram of body weight is considered fatal. High doses of peppermint oil are declared in animals to cause* * cystic spaces in cerebellar white matter and nephropathy in male rats [1]. In the same study, all doses of peppermint oil given to rats caused vacuolization of hepatocytes and increased liver weights. However, no sign of encephalopathy was observed [2]. On the other hand, excessive amount of menthol has been suggested to cause vertigo, dizziness, agitation, nystagmus, ataxia, hallucinations, lethargy, and coma [3].

Menthol has been shown to have analgesic, cooling, and muscle relaxing activities by its effect on transient receptor potential cation channel subfamily M member 8 (TRPM8), Kappa receptors stimulation, and inhibition of voltage gate sodium channels [4]. It enhances smooth muscle relaxation, reduces lower esophageal sphincter tone, and reduces the skin barrier by vasodilation [5, 6]. Adverse reactions to enteric-coated peppermint oil capsules are rare but can include hypersensitivity reaction, contact dermatitis, abdominal pain, acid reflux disease, perianal burning, decrease gastric emptying, bradycardia, and muscle tremor [1, 7, 8]. Twelve patients reported having oral ulceration, lichenoid reactions, and burning mouth syndrome after exposure to peppermint or menthol [9]. In mice, menthone, a structural substance related to menthol, was suggested to be involved in dopamine promoting ambulation [10]. No chronic exposure studies are reported in humans.

## 4. Conclusion

In general, most over the counter preparations are considered safe for general use; however, detailed dietary and herbal history is important which could lead to the diagnosis in unexplained and vague clinical presentation.

## References

[1] Spindler P, Madsen C (1992). Subchronic toxicity study of peppermint oil in rats. *Toxicology Letters*.

[2] Andersen FA (2001). Final report on the safety assessment of Mentha Piperita (Peppermint) Oil, Mentha Piperita (Peppermint) Leaf Extract, Mentha Piperita (Peppermint) Leaf, and Mentha Piperita (Peppermint) Leaf Water. *International Journal of Toxicology*.

[3] Opdyke DLJ (1976). Monographs on fragrance raw materials. *Food and Cosmetics Toxicology*.

[4] Gaudioso C, Hao J, Martin-Eauclaire MF, Gabriac M, Delmas P (2012). Menthol pain relief through cumulative inactivation of voltage-gated sodium channels. *Pain*.

[5] Hiki N, Kaminishi M, Yasuda K (2011). Multicenter phase Ii randomized study evaluating dose-response of antiperistaltic effect of L-menthol sprayed onto the gastric mucosa for upper gastrointestinal endoscopy. *Digestive Endoscopy*.

[6] Brain KR, Green DM, Dykes PJ, Marks R, Bola TS (2005). The role of menthol in skin penetration from topical formulations of ibuprofen 5% in vivo. *Skin Pharmacology and Physiology*.

[7] O’Mullane NM, Joyce P, Kamath SV (1982). Adverse CNS effects of menthol-containing olbas oil. *The Lancet*.

[8] GM N (1969). *Clinical Toxicology of Commercial Products*.

[9] Morton CA, Garioch J, Todd P, Lamey PJ, Forsyth A (1995). Contact sensitivity to menthol and peppermint in patients with intra-oral symptoms. *Contact Dermatitis*.

[10] Umezu T (2009). Evidence for dopamine involvement in ambulation promoted by menthone in mice. *Pharmacology Biochemistry and Behavior*.

